# The global burden of pressure ulcers among patients with spinal cord injury: a systematic review and meta-analysis

**DOI:** 10.1186/s12891-020-03369-0

**Published:** 2020-05-29

**Authors:** Wondimeneh Shibabaw Shiferaw, Tadesse Yirga Akalu, Henok Mulugeta, Yared Asmare Aynalem

**Affiliations:** 1 Department of Nursing, College of Health Science, Institute of Medicine and College of Health Science, Debre Berhan University, P.O. Box 445, Debre Berhan, Ethiopia; 2 Department of Nursing, College of Health Science, Debre Markos University, Debre Markos, Ethiopia

**Keywords:** Pressure ulcers, Spinal cord injury, Systematic review, Meta-analysis, Ethiopia

## Abstract

**Background:**

Pressure ulcers (PU), one of the common challenging public health problems affecting patient with spinal cord injury. PUs occurs over bony areas of the body where pressure and tissue distortion is greatest. It has a significant impact to the patient and health care system. Moreover, it has psychological, physical, social burden and decrease the quality of life (QoL) of patients. Despite its serious complications, limited evidence is available on the global magnitude of pressure ulcers among patient with spinal cord injury. Hence, this review and meta-analysis aimed to estimate the global magnitude of pressure ulcers among patient with spinal cord injury.

**Methods:**

PubMed, Scopus, Google Scholar, African Journals Online, PsycINFO, and Web of Science were systematically searched to retrieve related articles. The Preferred Reporting Items for Systematic Review and Meta-Analysis (PRISMA) guideline was followed. DerSimonian and Laird random-effects model was applied to obtain the pooled effect size. To investigate heterogeneity across the included studies, I^2^ test was employed. Publication bias was examined using funnel plot and Egger’s regression test statistic. Sensitivity analysis was deployed to see the effect of a single study on the overall estimation. Analysis was done using STATA™ Version 14 software.

**Results:**

A total of 24 studies which comprises of 600,078 participants were included in this study. The global pooled magnitude of pressure ulcers among patients with spinal cord injury was 32.36% (95% CI (28.21, 36.51%)). Based on the subgroup analysis, the highest magnitude of pressure ulcer was observed in Africa 41.19% (95% CI: 31.70, 52.18).

**Conclusion:**

This systematic review and meta-analysis revealed that about one in three patients with spinal cord injury had pressure ulcers. This implies that the overall global magnitude of pressure ulcer is relatively high. Therefore, policy maker and other concerned body should be design country context- specific preventive strategies to reduce the burden of pressure ulcers in patients with spinal cord injury.

## Background

Spinal cord injury (SCI) is a life-threatening and debilitating injury with tremendous immediate and long-term extensive impact on the medical, social, psychological and economic aspects of clients, their caregivers and the society [1–3]. The annual incidence rate of SCI is 44 cases per 1, 000, 000 people in Tehran [4], and 5.5 to 195.4 cases per million in European countries [5]. Spinal cord injured patients have a high risk of developing pressure ulcers due to motor and sensory impairments, immobility, changes in skin composition, and prolonged hospital stays [6, 7]. PUs are a serious, costly, and life-long complication of SCI. Around, 30–40% of clients with spinal cord injuries develop pressure ulcers during the acute and rehabilitation phases [8].

Pressure ulcer and its treatment is one of the most challenging clinical problems in hospitals specially among patients with spinal cord injury [9]. Even though several pressure ulcer classification systems are available, National Pressure Ulcer Advisory Panel (NPUAP) is the commonest method. According to the NPUAP consensus development conference, pressure ulcers are classified based on severity from suspected deep tissue injury through unstageable, with suspected deep tissue injury representing the earliest stage of pressure ulcer formation, and unstageable is defined as “full thickness tissue loss in which the base of the ulcer is covered by slough (yellow, tan, gray, green, or brown) and/or eschar (tan, brown, or black) in the wound bed” [10].

Though PU is largely preventable patient safety problems, they have major impacts to the affected individual and on the health care system. It highly affects the psychological, physical, social well-being and the quality of life of the affected individuals [11–13]. Likewise, it leads to recurrent hospitalizations, multiple surgeries, potentially devastating complications, increased costs for patient care, morbidity and early mortality [9, 14, 15]. PU carry a significant economic burden, with treatment costs of a PU far exceeding preventive costs. For example, a study done in Canada, revealed that the total monthly cost for treatment of PU among SCI was 18,758 USD [3]. Pressure ulcer may account for 25% of the overall cost of treating paraplegic and tetraplegic persons [16]. Moreover, a study done in Canada showed average monthly cost for treatment of PU in patients with SCI was $4745 [17].

Large differences have been reported on the magnitude of PUs patients with SCI in different studies. For instance, it varying from 11 to 50% in the current publications [6, 18]. Similarly, a study done in Switzerland reported that the incidence of PU was 2.31 per patient-year [18]. Poor pressure relief practices lead to PU development in persons with SCI [10]. Management and care of PU has become a serious public health challenge, with longer hospital stays than for other causes. Preventive measures to decrease the development of PUs consisted of basic skin care, pressure dispersion using fenestrated foams and alternating weight-bearing sites by regular frequent positioning [7]. In addition, the key targets for interventions has been advocated to reduce the burden of pressure ulcers in patient with SCI. These interventions includes identification of risk factors, patient education, acute intensive care, and support body surfaces [19].

The etiology of PUs is complex and rooted in multiple risk factors. Studies suggest that risk factors for PU in patient with SCI include duration after SCI (> 1 year) [20], age (older age), sex (being male) [20, 21], poor nutritional status [22], quadriplegia [23–25], smoking [6, 26], comorbidity [23, 27], severe Braden scores [28], weight (being underweight) [26], lower level of education [20, 21], and lack of an intimate partner [21] were some of the risk factors associated with PU. Similarly, it has been reported that patients with higher-level spinal cord injuries are more susceptible than those with lower-level lesions [13]. Hence, identification of risk factors used as benchmarks to design appropriate prevention measure, to improve client safety and efficient utilization of resources.

PUs among SCI clients remains unrelenting problem and a major issue in nursing care across the globe. Prevention of PU is a key role of nurses, and is considered a quality indicator of nursing care [29]. Thought most previous studies have been conducted, to assess the magnitude of PU in acute care setting, intensive care unit, and on public hospitals; however the global magnitude of PU among patient with SCI remains unknown. Hence, this study aimed to estimate the global burden of PU among spinal cord injured patients. Findings from the current study could be serve as benchmark for institutional health care policy-makers to implement appropriate preventive measures to reduce and prevent PU. In addition, it will assist clinicians in estimating burden of PU as part of an overall quality indicator for facilities and an assessment of the effectiveness prevention strategies.

## Methods

### Search strategy and database

A two-step search strategy was used to identify all relevant literature. First, six electronic databases such as PubMed, Google Scholar, Africa Journals Online, Scopus, Web of Science, and PsycINFO were systematically searched. Second, a manual search of grey literatures was performed to identify additional relevant research to augment our meta-analysis. All electronic sources of information were searched for the period of January 1, 2000 to July 2, 2019. The search was conducted using the following terms and phrases: “pressure ulcer”, “pressure injury”, “decubitus ulcer”, “spinal cord injury”, and “prevalence”. Boolean operators like “AND” and “OR” were used to combine search terms.

### Eligibility criteria

Studies were included if they fulfilled the following criteria: (1) All observational studies which report the prevalence PU among SCI; (2) articles published in peer reviewed journals and grey literature:(3) published in the English language between 2000 to 2019; (4) we imposed no restriction on the area where it is conducted; and (5) the group of patients admitted without PUs. Studies were excluded on any one of the following conditions: (1) Patients admitted with PU; (2) they have poor quality score as per the stated criteria; and (3) articles in which fail to estimate the outcome (PU).

### Selection and quality assessment

Data were extracted by three authors using a pre-piloted and standardized data extraction format prepared in a Microsoft excel. For each included study, the following data were extracted: the name(s) of the author (‘s), year of publication, study area, study design, sample size, prevalence, data collection year, and data collection methods. The quality of each included study was assessed using the Newcastle-Ottawa scale (NOS). Articles were included in the analysis if they scored ≥7 out of 10 points in three domains of the equally weighted modified NOS components for a cohort studies [30], (Additional file 1). In addition, for cross-sectional studies quality assessment tool, score of ≥5 out of 10 considered as high quality score [31], (Additional file 2). On the other hand, to assess the risk of bias within studies tools developed for observational studies by Hoy and colleagues was used [32]. The tool has ten items to assess the risk of bias (i.e. external validity has four items and internal validity has six items). Based on these items the overall risk of study bias could be summary as low risk of bias, moderate risk of bias, and high risk of bias. The quality score of each study were extracted from each incorporated article by three independent authors. Any disagreements during data extraction were resolved by discussion and consensus.

### Statistical analysis

To obtain the meta-weighted burden of pressure ulcer among SCI, a meta-analysis using random-effects model DerSimonian and Laird method was employed. Cochran’s Q chi-square statistics and I^2^ statistical test was conducted to assess the random variations between primary studies [33]. In case of high heterogeneity, subgroup analysis to reduce heterogeneity, and meta regression analysis to explore sources of heterogeneity were utilized. Publication bias was assessed by visual inspection of a funnel plot and objectively using the Egger bias test [34, 35]. Sensitivity analysis was performed to investigate whether the pooled effect size was influenced by individual studies. The data were analysed using STATA™ Version 14 statistical software [36].

### Data synthesis and reporting

The results of this review were reported based on the Preferred Reporting Items for Systematic Reviews and Meta-Analyses (PRISMA) guideline was followed [37]. Moreover, results were presented using forest plots and summary tables.

## Result

### Search results

**Fig. 1:**
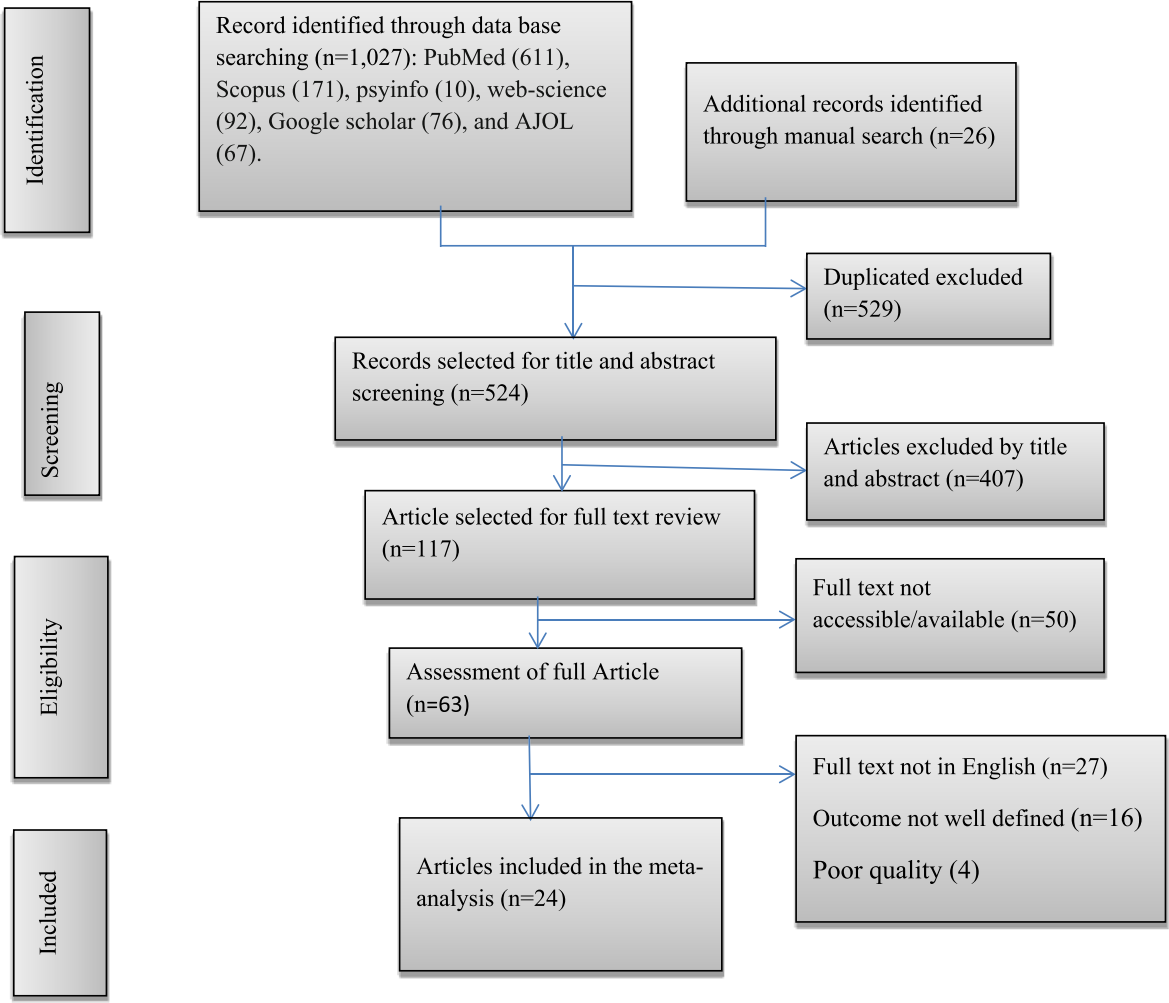
PRISMA flowchart diagram of the study selection

We found a total of 1053 articles. Of these, 1027 studies were found from PubMed (611), Scopus (171), PsycINFO (10), Google Scholar (76), Web of Science (92), and Africa Journals Online (67). In addition, the remaining 26 were retrieved through manual search. Of these, 529 articles were excluded due to duplication. Following the removal of duplicate studies, the titles and abstracts were evaluated, and 407 studies were excluded after reading of titles and abstracts base on the pre-specified inclusion criteria. Then, a total 117 full text articles were assessed for their eligibility based on the pre-set inclusion criteria. After reviewing the full text, based on the pre-defined criteria and quality assessment, 24 articles were included for the final analysis (Fig. 1).

### Baseline characteristic of the included studies

A total of 24 studies with 600,078 study participants were included to estimate the pooled prevalence of PU among SCI patients. In this meta-analysis, various countries were represented across a globe: 8 of studies were from America [6, 38–43], 7 from Europe [18, 23, 44–48], 5 from Asia [20, 28, 49–51], and 4 from Africa [7, 22, 24, 52]. The highest prevalence of PU (56%) was reported from Europe [44], whereas the lowest prevalence (11%) was reported in a study conducted in America [6]. With respect to sample size, the number of study participants varied from 38 to 7489. The quality score of each primary study, based on the Newcastle- Ottawa quality score assessment, was moderate to high for all 24 articles assessed (Table 1).

**Table 1:** Demonstrates the baseline characteristic of primary studies

First Author	Pub. year	study country, continent	study design	sample size	Prevalence% (95%CI)	Data Collection year	data collection methods	Quality score
Ash, D etal [44]	2002	United Kingdom, Europe	Cross-sectional	144	56(47.9,64.1)	1998 to 2000	document review	7
Brienza,D., et al. [38]	2017	United States, North America	cohort	104	37.5(28.2,46.8)	2008–2012	observation and examination	7
Chopra,T.,et al. [53]	2016	United States, North America	Cross-sectional	201	38(31.3,44.7)	January 2004 and December 2008	document review	7
DeJong, G., et al. [39]	2014	United states, north America	Cohort	159	13.1(7.8,18.3)	NA	Document review	7
Eslami V et al. [20]	2012	Iran, Asia	Cross-sectional	7489	34.6(33.5,35.7)	June 2007 to June 2009	physical examination	9
Fazel FS, etal [28]	2018	Iran,Asia	cohort	580	28.1(24.4,31.7)	June 2013 to December 2015	Observation and examination	8
Garber, S.L., et al. [40]	2000	United states, North America	Cohort	118	31(22.2,39.3)	NA	Interview and exam	8
Haisma, J.A., et al. [45]	2007	Netherlands,Europe	cohort	212	36(29.5,42.5)	May 2000 and September 2003	Self-report	8
Idowu, O., et al. [22]	2011	Nigeria,Africa	cohort	105	45.9(36.4,55.4)	1 October 2004 and 30November 2006	Skin examination	8
Iyun A.O. etal [7]	2012	Nigeria, Africa	cohort	67	47.7(35.7,59.7)	January 2003 to June 2004	Self-report and documentation	7
Joseph, C. and L.N. Wikmar [24]	2015	south Africa,Africa	cohort	141	29.8(22.2,37.4)	15 September 2013 to 14 September 2014	observation and examination	8
Kovindha, A. et al. [49]	2015	Thailand, Asia	Cross-sectional	129	26.4(18.8,34)	1 January 2013 to 31December 2013	Self-report	7
Krishnan,S., et al. [41]	2017	United States, North America	Cross-sectional	3098	20.3(18.9,21.7)	1993 to 2006	document review	8
Li,C., et al. [6]	2016	United States,North America	Cross-sectional	350	11(7.6,14.4)	August 2011 to February 2014	Self-report	8
Löfvenmark I et al. [52]	2016	Botswana,Africa	cohort	38	48(32.1,63.9)	1February 2011 to 31January 2013	Skin examination	8
Raghavan, P., et al., [46]	2003	United kingdom, Europe	Cross-sectional	472	23(19.2,26.8)	NA	Observation and exam	7
Richard-Denis, A., et al. [42]	2016	Canada,North America	Cohort	123	33.3(24.9,41.6)	January 1, 2009, and December 31, 2011	Document review	7
Saunders et al. [43]	2013	United States, North America	Cross-sectional	2549	19.9(18,21.7)	NA	mail-in survey	9
Scheel-Sailer, A., et al. [47]	2013	Switzerland,Europe	cohort	185	25.4(19.1,31.7)	1 September 2009 to 28February 2010	observation and examination	7
Sheerin, F. etal [48]	2005	Ireland, Europe	Cross-sectional	82	37(26.5,47.4)	December 2000 to December 2002	document review	7
Taghipoor, K.D., et al. [51]	2009	Iran, Asia	Cross-sectional	3791	39.1(37.5,40.6)	NA	Document review	7
Tchvaloon,E., et al. [50]	2007	Israel, Asia	cohort	143	26.6(19.4,33.8)	1962 and 2004	document review	7
van der Wielen H et al. [18]	2016	Switzerland,Europe	cohort	185	50(42.8,57.2)	September 2009 to February 2010	observation	7
Verschueren J et al. [23]	2011	Netherlands, Europe	cohort	193	36.5(29.7,43.3)	NA	observation and examination	8

*N/A* not applicable

### Global burden of pressure ulcer among patients with spinal cord injury

**Fig. 2:**
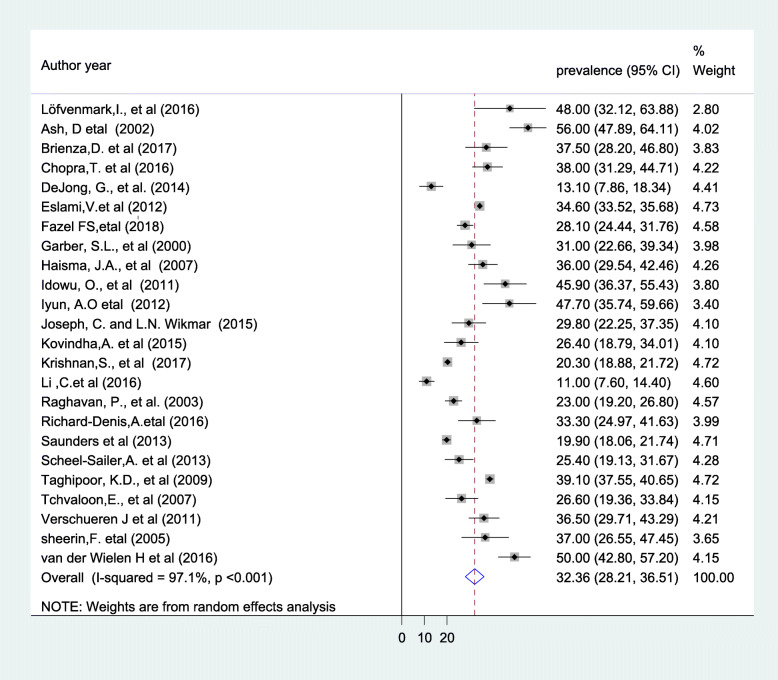
The global pooled prevalence of PU in patients with SCI

The finding of current meta-analysis using a random effects model indicated that the global pooled magnitude of PU in patients with SCI was found to be 32.36% (95% CI: 28.21–36.51), (Fig. 2) with a substantial level of heterogeneity (I^2^ = 97.1%; *P* < 0.001).

### Sub-group analysis

**Fig. 3:**
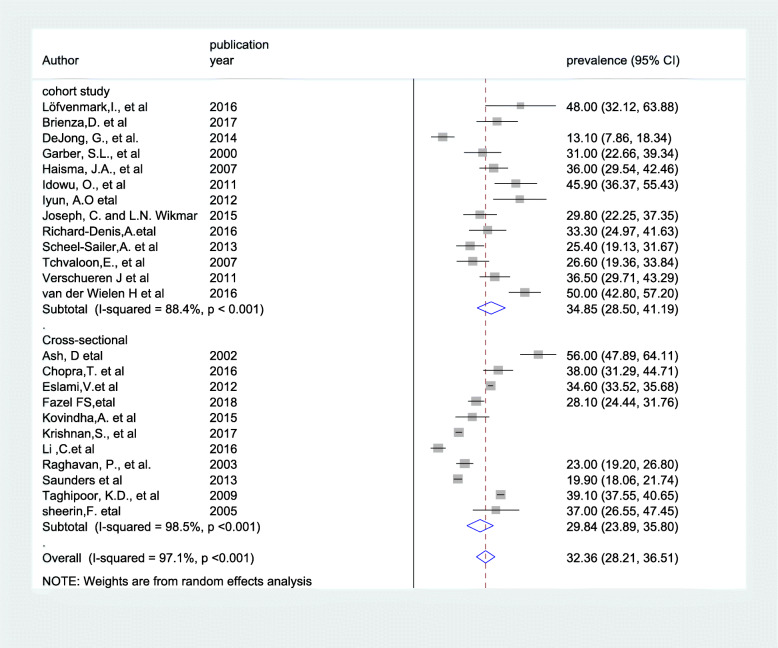
Forest plot showing subgroup analysis by study design

**Fig. 4:**
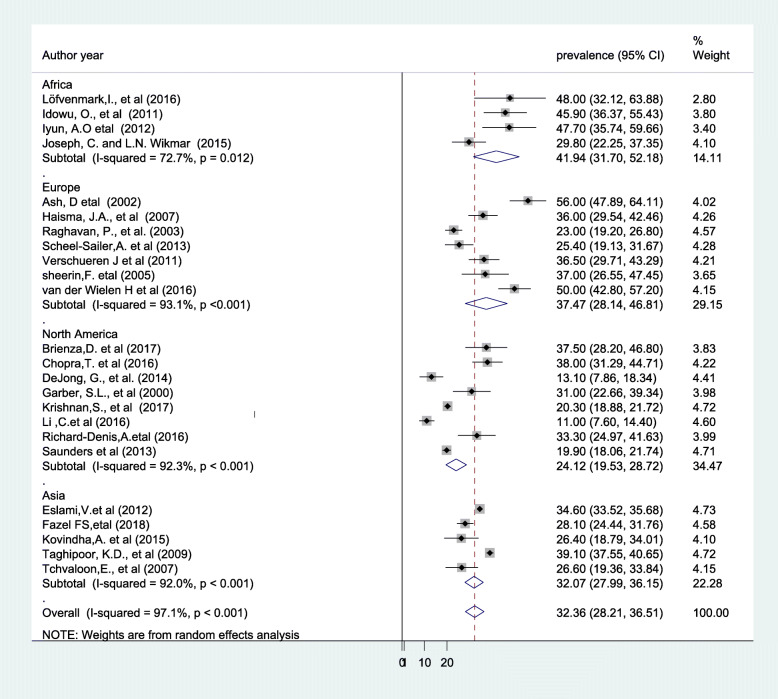
Forest plot showing subgroup analysis by study continent

The presence of significant heterogeneity among the primary studies requires the need to conduct subgroup analysis. As a result, the finding of subgroup analysis using study design showed that the highest magnitude of pressure ulcer was observed among study done using cohort design which was 34.85% (95% CI: 28.50, 41.19), I^2^ = 88.4%) (Fig. 3). Concerning with study continent, high magnitude of pressure ulcers was observed among study done in Africa which was 41.94%(95% CI: 31.70, 52.18), I^2^ = 72.7%) of our included primary studies, (Fig. 4).

### Meta- regression analysis

In the present review, we conducted meta-regression analysis by considering year of publication, and sample size as covariates of interest to identify the possible sources of random variations across primary studies. But, the result of the meta-regression analysis showed that both covariates were not statistically significant source of heterogeneity (Table 2).

**Table 2:** Meta regression analysis for the included studies to identify source of heterogeneity

Covariate (source)	Coefficient	Standard error	*P*-value	95% Conf. Interval
Publication year	−0.416	0.530	0.442	− 1.526, 0.694
Sample size	0.0002	0.001	0.869	−0.003, 0.0035

### Publication bias

**Fig. 5:**
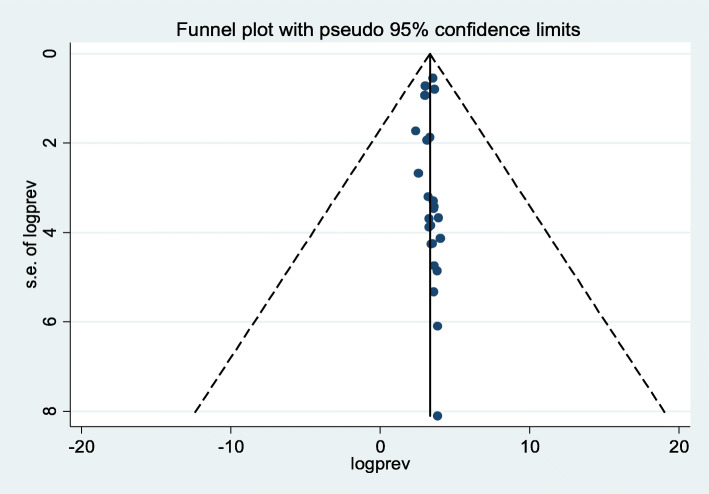
Funnel plot to test the presence of publication bias of the 24 studies

To identify the presence of publication bias funnel plot, and Egger’s regression test were performed. Therefore, we obtained that there was no publication bias among the included studies, as illustrated by the symmetrical distribution of a funnel plot (Fig. 5). Likewise, Egger’s regression test results, showed that there was no statistically significant publication bias across the included studies as evidenced by (*P* = 0.74).

### Sensitivity analysis

**Fig. 6:**
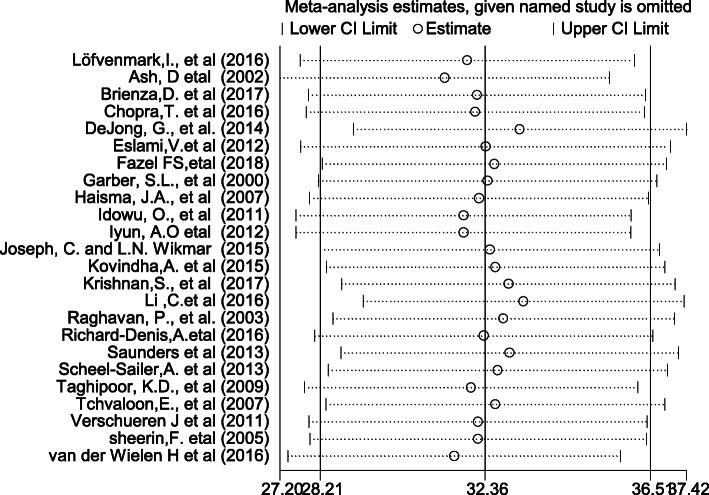
Result of sensitivity analysis of the 24 studies

The finding of sensitivity analysis using random effects model revealed that no single study affected the overall magnitude of pressure ulcer among SCI (Fig. 6).

## Discussion

The current meta-analysis provided up-to-date knowledge on the magnitude of PU among spinal cord injured patients. The present findings revealed that the global pooled prevalence of PU in patients with SCI was 32.36% (95% CI: 28.21, 36.51%). This finding is much higher than a meta-analysis study done on global prevalence of PU in public hospitals at14.8% [54]. This indicated that PU is highly prevalent among patients with spinal cord injury, and reflect an inadequately prevention and management of PU risk factors.

The result of the subgroup analysis based on study design (cross-sectional vs cohort) showed that the highest pooled prevalence of PU was observed from those studies with cohort design 34.85% (95%CI: 28.50, 41.19). This variation might be in case of cohort design the outcome variable was obtained through observation, skin assessment, physical examination, and with certain follow up time, whereas in cross-sectional studies data were collected with document review and self-report. Therefore, these situations may contribute for variation across study designs. Similarly, the result of subgroup analysis based on continent revealed that the highest magnitude of PU was reported in Africa 41.94%, followed by Europe 37.47%. The possible explanations for this variation might be due methodological differences, variation in quality of care, differences in health seeking behavior between the populations, policy and strategy difference [9, 55].

This study has clinical implications in that the increase burden of PU in SCI patients should alarm policy makers and clinician to enhance patient awareness on the impact of PU, to adhere preventive measures, to improve patient safety, to minimize treatment cost, and to design treatment strategies for PU among patients with SCI. In addition, the finding serves as a bench mark to health care professional to provide a attention on the application of standardized care, and represents a marker of quality of care.

The current review and meta-analysis has some limitations: First, inclusion of English only articles, when this was international based review; Second, this study do not identify the predictors of pressure ulcers among patients with spinal cord injury; Third, it may not be representative of the entire content, as no data were found form all of the globe; and fourth, all included studies were reported hospital-based data.

## Conclusion

This systematic review and meta-analysis revealed that about one in three patients with spinal cord injury had pressure ulcers. This implies that the overall global magnitude of pressure ulcer is relatively high. Therefore, policy makers and other concerned body should be design country context specific effective preventive strategies to decrease the burden of PU among patients with SCI, and to improve the overall quality of healthcare service at large. Furthermore, further research is needed to identify associated factors for the development of PU among patient with SCI.

## Supplementary information

**Additional file 1.** Methodological quality assessment of cohort studies using modified Newcastle - Ottawa Scale (NOS).

**Additional file 2.** Methodological quality assessment of cross-sectional studies using modified Newcastle - Ottawa Scale (NOS).

## Data Availability

The data analyzed during the current meta-analysis is available from the corresponding author on reasonable request.

## Abbreviations

CI: Confidence Interval
NPUAP: National Pressure Ulcer Advisory Panel
PU: Pressure Ulcer
PRISMA: Preferred Reporting Items for Systematic Reviews and Meta-Analyses
SCI: Spinal Cord Injury
