# Supplementary material for: The global burden of pressure ulcers among patients with spinal cord injury: a systematic review and meta-analysis
Source: BMC Musculoskelet Disord. 2020 May 29;21:334. doi: 10.1186/s12891-020-03369-0 (PMC7260823; doi:10.1186/s12891-020-03369-0)
Supplement: Supplementary file 1 — Additional file 1. Methodological quality assessment of cohort studies using modified Newcastle - Ottawa Scale (NOS). [file 12891_2020_3369_MOESM1_ESM.docx]

Additional file 1: Methodological quality assessment of cohort studies using modified Newcastle - Ottawa Scale (NOS)

| **First author, publication year** | Criteria | | | | | | | |  |
| --- | --- | --- | --- | --- | --- | --- | --- | --- | --- |
|  | **Selection** | | | |  | **Outcome** | | |  |
|  | **Representativeness of exposed cohort** | **Representativeness of non- exposed cohort** | **Incident disease** | **Ascertainment of exposure** | **Comparability** | **Length of follow up** | **Assessment of the outcome** | **Adequacy of follow up** | **Total score**  **(9)** |
| Haisma, J.A., et al 2007 | **A*** | **B*** | **A*** | **A*** | **A*** | **A*** | A* | **A*** | **8** |
| van der Wielen H et al 2016 | **A*** | **B*** | **A*** | **A*** | **A*** | **B*** | A* | **A*** | **7** |
| Verschueren J et al 2011 | **A*** | **B*** | **A*** | **B*** | **A*** | **A*** | A* | **A*** | **8** |
| Löfvenmark I et al,2016 | **B*** | **A*** | **A*** | **B*** | **A**** | **A*** | A* | **A*** | **8** |
| Iyun A.O. etal 2012 | **A*** | **B*** | **A*** | **A*** | **C** | **B*** | A* | **A*** | **7** |
| Brienza,D., et al 2017 | B* | **B*** | **A*** | **A*** | **A*** | **C** | A* | **A*** | **7** |
| Scheel-Sailer, A., et al 2013 | A* | **B*** | **A*** | **A*** | **A*** | **A*** | A* | **A*** | **7** |
| Idowu, O., et al 2011 | A* | **A*** | **A*** | **A*** | **A**** | **A*** | B* | **A*** | **8** |
| Tchvaloon,E., et al 2007 | A* | **B*** | **A*** | **A*** | **A*** | **A*** | B* | **A*** | **7** |
| Joseph, C. and L.N. Wikmar 2015 | A* | **A*** | **A*** | **A*** | **A*** | **B*** | A* | **A*** | **8** |
| Fazel FS, etal 2018 | B* | **B*** | **A*** | **A*** | **A**** | **A*** | A* | **A*** | **8** |
| Richard-Denis, A., et al 2016 | A* | **B*** | **A*** | **C** | **A*** | **B*** | A* | **A*** | **7** |
| DeJong, G., et al.2014 | B* | **B*** | **A*** | **A*** | **A*** | **A*** | A* | **A*** | **7** |
| Garber, S.L., et al,2000 | A* | **B*** | **A*** | **A*** | **A**** | **A*** | A* | **A*** | **8** |

*Note: from each item account point. (Accept the study if total score ≥7)*
