# Supplementary material for: The global burden of pressure ulcers among patients with spinal cord injury: a systematic review and meta-analysis
Source: BMC Musculoskelet Disord. 2020 May 29;21:334. doi: 10.1186/s12891-020-03369-0 (PMC7260823; doi:10.1186/s12891-020-03369-0)
Supplement: Supplementary file 2 — Additional file 2. Methodological quality assessment of cross-sectional studies using modified Newcastle - Ottawa Scale (NOS). [file 12891_2020_3369_MOESM2_ESM.docx]

Additional file 2: Methodological quality assessment of cross-sectional studies using modified Newcastle - Ottawa Scale (NOS)

| **First author, publication year** | Criteria | | | | | | | |  |
| --- | --- | --- | --- | --- | --- | --- | --- | --- | --- |
|  | **Selection** | | | | **Comparability** | | **Outcome** | |  |
|  | **Representativeness of the sample** | **Sample size** | **Non –respondents** | **Ascertainment of exposure/risk factor** | **The study controls for the most important factor** | **The study control for any additional factor** | **Assessment of the outcome** | **Statistical test** | **Total score**  **(10)** |
| Saunders et al 2013 | **A*** | **A*** | **A*** | **A**** | **B*** | **A*** | A* | **A*** | **9** |
| Ash, D etal 2002 | **A*** | **A*** | **A*** | **A*** | **A*** | **B*** | A* | **A*** | **7** |
| Eslami V et al 2012 | **A*** | **A*** | **A*** | **A*** | **A*** | **B*** | A** | **A*** | **9** |
| Krishnan,S., et al 2017 | **A*** | **A*** | **A*** | **B*** | **A*** | **A*** | A* | **A*** | **8** |
| Li ,C., et al ,2016 | **B*** | **A*** | **A*** | **B*** | **A*** | **A*** | A** | **A*** | **8** |
| Kovindha, A. et al 2015 | **A*** | **A*** | **A*** | **A*** | **-** | **B*** | A* | **A*** | **7** |
| Sheerin, F. etal 2007 | B* | **B*** | **A*** | **A*** | **A*** | **-** | A* | **A*** | **7** |
| Chopra,T.,et al 2016 | A* | **A*** | **A*** | **A*** | **A*** | **-** | A* | **A*** | **7** |
| Taghipoor, K.D., et al 2009 | A* | **A*** | **C** | **A*** | **A*** | **A*** | A* | **A*** | **7** |
| Raghavan, P., et al.,2003 | A* | **A*** | **A*** | **A*** | **A*** | **B*** | A* | **A*** | **7** |

*Note: from each item account point. (Accept the study if total score ≥5)*

Selection: (Maximum 5 stars)
1) Representativeness of the sample: a) Truly representative of the average in the target population. * (all subjects or random sampling) .b) Somewhat representative of the average in the target population. * (nonrandom sampling) .c) Selected group of users.d) No description of the sampling strategy.
2) Sample size:a) Justified and satisfactory. *.b) Not justified.
3) Non-respondents: a) Comparability between respondents and non-respondents characteristics is
established, and the response rate is satisfactory. * .b). The response rate is unsatisfactory, or the comparability between respondents
and non-respondents is unsatisfactory. c) No description of the response rate or the characteristics of the responders and
the non-responders.
4) Ascertainment of the exposure (risk factor): a) validated measurement tool. ** .b) Non-validated measurement tool, but the tool is available or described.* c) No description of the measurement tool.
Comparability: (Maximum 2 stars)
1) The subjects in different outcome groups are comparable, based on the study design or analysis. Confounding factors are controlled. a) The study controls for the most important factor (select one). * b) The study control for any additional factor. *
Outcome: (Maximum 3 stars)
1) Assessment of the outcome: a) Independent blind assessment. **,b) Record linkage. **,c) Self report. *,d) No description.
2) Statistical test:a) The statistical test used to analyze the data is clearly described and appropriate, and the measurement of the association is presented, including confidence intervals and the probability level (p value). *,b) The statistical test is not appropriate, not described or incomplete
